# Left-Leg Apraxia and Bilateral Incoordination of the Lower Limb After Left Anterior Cerebral Artery Infarction: A Case Report

**DOI:** 10.7759/cureus.82055

**Published:** 2025-04-11

**Authors:** Airi Kitamura, Hiroyuki Ohtsuka, Miku Aoyagi, Maho Noguchi, Naoyuki Motojima, Kana Sakuma, Tetsuichi Hondera, Mika Otsuki

**Affiliations:** 1 Department of Physical Therapy, Showa Medical University, Yokohama, JPN; 2 Graduate School of Health Sciences, Showa Medical University, Yokohama, JPN; 3 Department of Rehabilitation Medicine, Showa Medical University, Yokohama, JPN; 4 Institute of Clinical Epidemiology, Showa Medical University, Yokohama, JPN; 5 Center for Rehabilitation, Showa Medical University, Yokohama, JPN; 6 Department of Rehabilitation Medicine, Showa Medical University, Tokyo, JPN; 7 Division of Health Science Education, Showa Medical University, Yokohama, JPN; 8 Graduate School of Health Sciences, Hokkaido University, Sapporo, JPN

**Keywords:** bilateral lower-limb coordination, callosal disconnection syndrome, cerebral infarction, corpus callosum, interhemispheric communication, leg apraxia, motor coordination, rehabilitation

## Abstract

Callosal disconnection syndrome is characterized by impaired interhemispheric communication and motor coordination. Although its effects on upper limbs are well documented, reports on lower-limb function are limited, and their pathological mechanisms remain poorly understood. We describe a case involving a right-handed woman in her early 70s who presented with slurred speech and difficulty moving her feet. Magnetic resonance imaging revealed an atherothrombotic cerebral infarction affecting the splenium, body, and genu of the corpus callosum, as well as the medial region of the left frontal lobe. In addition to callosal disconnection symptoms, including impaired transfer of proprioceptive information from the fingers, left-hand apraxia, and intermanual conflict, this patient exhibited asynchrony in lower-limb movements and left lower-limb apraxia. The patient’s ability to perform coordinated movements improved with repeated rehabilitation sessions. These findings suggest that corpus callosum injury plays a pivotal role in bilateral lower-limb coordination and motor control. We emphasize the need for further research to elucidate the role of the corpus callosum in lower-limb motor control.

## Introduction

The corpus callosum, the largest commissural fiber connecting the two cerebral hemispheres, serves as a critical structure supporting the integration of motor, sensory, and cognitive functions [1]. Its function is largely dependent on an adequate blood supply from the anterior cerebral artery (ACA), which primarily perfuses the anterior and midbody regions. Consequently, infarctions in this territory can significantly disrupt interhemispheric communication [2]. Callosal disconnection syndrome is widely recognized as a condition characterized by impaired information transfer and interhemispheric coordination [3]. Most published reports have focused on upper-limb effects, whereas lower-limb symptoms remain sparsely described [4].

While callosal dysfunction has been widely reported to impair bimanual coordination in the upper limbs [5], its role in lower-limb motor control remains less well understood. Given the extensive interhemispheric connections involved in lower-limb motor function, corpus callosum lesions may disrupt lower-limb coordination and motor planning. Recent advances in tractography have revealed that the anatomical connections between the bilateral hemispheric lower-limb motor areas are prominently organized within the body of the corpus callosum [6]. Furthermore, in patients with multiple sclerosis, a relationship between callosal inhibitory dysfunction and lower-limb motor impairment has been postulated [7,8]. Thus, corpus callosum lesions may impact motor control of the lower limbs in a manner similar to their impact on the upper limbs. However, available evidence remains limited, and only a few studies have explored the specific effects of corpus callosum damage on lower-limb motor control.

Damage to the corpus callosum disrupts interhemispheric motor communication and can impair the execution of learned motor actions, leading to ideomotor apraxia. In right-handed individuals, callosal lesions commonly result in left upper-limb ideomotor apraxia due to impaired transfer of motor engrams from the dominant left hemisphere to the right motor areas [9]. While ideomotor apraxia is well documented in the upper limbs, recent studies suggest that it can also affect the lower limbs, though less frequently reported [10]. Lower-limb ideomotor apraxia, similar to upper-limb apraxia, has been shown to impair gesture imitation, particularly in patients with left hemisphere lesions, suggesting a shared neural mechanism for praxis across the limbs [10]. This shared mechanism supports the idea that the corpus callosum, a key structure in interhemispheric motor integration, may similarly contribute to lower-limb ideomotor apraxia, although its precise mechanisms remain unclear.

In this case report, we describe the clinical findings and provide insights into the pathological mechanisms in a patient with infarctions affecting the corpus callosum and the region perfused by the left ACA, which impaired bilateral lower-limb coordination and induced ideomotor apraxia in the left lower limb.

## Case presentation

A timeline summarizing the patient’s clinical course, including key assessments and interventions from the acute hospitalization to the rehabilitation phase, is provided in the appendix.

**Figure 1:**
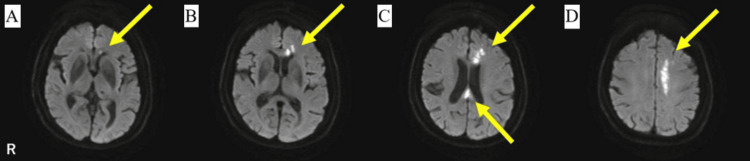
Diffusion-weighted MR images obtained 7 days after symptom onset (A-D) High-signal intensity regions are visible in the splenium, body, and genu of the corpus callosum (yellow arrows). MR: magnetic resonance

A right-handed woman in her early 70s with a history of hypertension presented to the emergency department on Day X with complaints of slurred speech and difficulty walking. She reported that on the morning of Day X−1, she began experiencing difficulty moving her legs. By the afternoon of Day X, she noticed slurred speech and decided to seek emergency care. Her symptoms included a sense of heaviness and weakness in the right leg, feelings of fear and instability, and difficulty lifting the leg, with expressions such as "my knee feels shaky" and "I can't lift my foot." She had been last seen in her normal state approximately 36 hours before hospital arrival. Subsequently, magnetic resonance imaging (MRI) revealed atherothrombotic infarctions affecting the splenium, body, and genu of the corpus callosum and the medial aspect of the left frontal lobe (Figure 1). Neurological examination revealed clear consciousness, no visual field defects, no facial nerve palsy, negative Barré sign, no ataxia, mild sensory impairment in the right foot, and mild word-finding difficulty. She did not receive thrombolytic therapy with tissue plasminogen activator (tPA) and was treated with antiplatelet therapy (aspirin 100 mg daily) during the acute phase and received rehabilitation for 12 days at an acute care hospital. Subsequently, the patient was transferred to a recovery-phase rehabilitation hospital for continued care. At the time of transfer, her functional independence measure (FIM) score was 44, comprising 24 points for motor function and 20 points for cognitive function. Neurological and neuropsychological examinations were conducted between 20 and 40 days after onset.

Consciousness was intact. The Hasegawa Dementia Scale-Revised (HDS-R) score was 21 out of 30, indicating mild cognitive impairment. No visual field defects or facial nerve palsy were observed, and the Barré sign was negative. Additionally, mild word-finding difficulty was noted. Deep tendon reflexes were hyperactive in the right biceps, triceps, patellar, and Achilles tendons. No motor paralysis was observed, with Brunnstrom Recovery Stages scores of VI for the upper limb, hand, and lower limb. No signs of cerebellar ataxia, involuntary movements, or other coordination deficits, such as dysmetria or intention tremors, were observed. The patient reported mild numbness in the right sole, but the deep sensation remained intact.

The neuropsychological assessment identified deficits in shifting attention, indicated by poor performance on Trail Making Test Part A (376 s) and perseveration, suggesting general attention impairment. Frontal lobe dysfunction was evident, with a score of 7/18 on the Frontal Assessment Battery. Impaired interhemispheric transfer of proprioceptive information from the fingers, left-hand ideomotor apraxia, and intermanual conflict were also observed. Speech and language assessments revealed aphasia, including word-finding difficulties, phonemic paraphasia, and acquired stuttering. Additionally, the Standard Performance Test for Apraxia (SPTA) revealed impairments across multiple domains, particularly in the left hand and left lower limb (Table 1). Severe errors were observed in habitual gestures, meaningless gestures, tool use, and bilateral coordination tasks, especially during imitation and action execution. Mild errors were also noted in figure copying, dressing, and lower-limb tool use. These findings indicate broader impairments in skilled movement execution and coordination, extending beyond upper-limb dysfunction to include lower-limb motor control.

**Table 1:** Progression of Standard Performance Test for Apraxia (SPTA) scores NE: not examined; *1: only two of the original one SPTA item were assessed in this case; *2: only three of the original four SPTA items were assessed in this case

		Initial assessment (Days 29-31)			Final assessment (Days 108-109)		
	Type of command	Number of error items		Number of total items	Number of error items		Number of total items
		Severe errors	Mild errors		Severe errors	Mild errors	
Face, habitual gesture	Verbal commands	1	0	3	0	0	3
	Imitation	0	1	3	0	0	3
Face, gesture of tool use	Verbal commands	0	0	1	0	0	1
	Imitation	0	0	1	0	0	1
Face, actual tool use	Verbal commands	0	0	1	0	0	1
	Imitation	0	0	1	0	0	1
Upper limb, habitual gesture	Right hand, verbal commands	0	1	3	0	0	3
	Right hand, imitation	1	0	3	0	0	3
	Left hand, verbal commands	2	1	3	0	1	3
	Left hand, imitation	1	1	3	0	1	3
Upper limb, meaningless gesture	Right hand, imitation	0	0	2	0	0	2
	Left hand, imitation	0	2	2	0	0	2
	From left to right, transfer	1	0	1	1	0	1
	From right to left, transfer	1	0	1	1	0	1
Both hands, meaningless gesture	Imitation	1	0	3	1	0	3
Upper limb, sequential meaningless gesture	Right hand, imitation	0	1	1	0	0	1
	Left hand, imitation	1	0	1	0	0	1
Upper limb, dressing	Verbal commands	0	1	1	NE	NE	1
	Imitation	0	0	1	NE	NE	1
Upper limb, gesture of tool use	Right hand, action commands	0	0	4	0	0	4
	Left hand, action commands	1	1	4	0	1	4
	Right hand, imitation	0	0	4	0	0	4
	Left hand, imitation	1	2	4	0	0	4
Upper limb, actual tool use	Right hand, use commands	0	0	4	0	0	3^*2^
	Left hand, use commands	1	1	4	0	1	3^*2^
	Right hand, action commands	0	0	4	0	0	3^*2^
	Left hand, action commands	1	1	4	0	1	3^*2^
	Right hand, imitation	0	0	4	0	0	3^*2^
	Left hand, imitation	0	2	4	0	0	3^*2^
Upper limb, sequential habitual gesture	Verbal commands	0	1	1^*1^	0	0	1^*1^
Lower limb, gesture of tool use	Right lower limb	0	0	1	0	0	1
	Left lower limb	0	1	1	0	0	1
Lower limb, actual tool use	Right lower limb	0	0	1	0	0	1
	Left lower limb	1	0	1	0	0	1
Figure drawing (without model)	Right hand	0	0	2	0	0	2
	Left hand	0	0	2	0	1	2
Figure drawing (copying)	Right hand	0	1	2	0	0	2
	Left hand	0	1	2	0	1	2
Building block test	Right hand	0	0	1	0	0	1
	Left hand	1	0	1	NE	NE	1

Impairments in bilateral lower-limb coordination were observed during two specific tasks. In the foot pat task, the patient performed actions separately with each foot but exhibited asynchronous and mismatched timing when instructed to operate both foot pats simultaneously, resulting in uncoordinated motion (Video 1). Significant rhythmic disruption was observed during stair descent. After one foot landed on a step, the other involuntarily landed on the same step, resulting in a need for assistance. During the ergometer task, the patient initially exhibited asynchronous leg movements and could not perform smooth reciprocal actions. Notably, one foot began pedaling immediately after the other completed its movement, demonstrating mismatched timing and difficulty in achieving smooth pedal rotation. Nevertheless, repeated sessions led to gradual improvement in coordinated reciprocal movements and motor rhythm. Notably, the patient reported a prior ability to ride a bicycle but had never used an ergometer before. To further evaluate lower-limb coordination without the influence of tactile stimulation or gravity, we assessed cycling motion in a supine position. This evaluation revealed an asymmetric movement pattern, with noticeable disruptions in rhythmic alternation between the lower limbs.

**Video 1:** Execution of the foot pat task In the initial portion of the video, the patient successfully presses the foot pat with each foot individually, demonstrating appropriate timing for the left and right feet. In the latter portion, when instructed to press the foot pat simultaneously with both feet, asynchronous movements and a lack of phase synchronization are observed.

Left-leg apraxia was evident during the evaluation process of the SPTA (Table 1). The SPTA was conducted in the following sequence: right lower-limb gesture kicking, left lower-limb gesture kicking, actual right lower-limb kicking, and actual left lower-limb kicking. The number of trials was limited to one or two. During the left lower-limb gesture task, when instructed to “pretend to kick a ball” in a seated position, the right foot performed a smooth kicking motion. However, the left foot failed to execute the expected movement and instead displayed simple knee flexion and extension, which was classified as a mild error (Video 2). Additionally, when the patient was instructed to perform an actual ball-kicking motion in a seated position, she incorrectly stepped on the ball instead of kicking it, leading to a severe error classification. Both tasks were performed normally by the right lower limb. Although reproducibility was not explicitly tested through repeated trials of the same task, a similar response pattern was observed in a concurrent motor assessment. When instructed to imitate left lower-limb flexion during muscle strength testing in a seated position, the patient initially exhibited an incorrect knee extension response. However, with repeated attempts, she gradually succeeded in performing the correct motion. Furthermore, during rehabilitation training, the patient was required to walk while kicking a semicircular ball. While the right foot successfully executed the instructed motion, the left foot inappropriately stepped on the ball, mirroring the errors observed during the SPTA.

**Video 2:** Observation of the “pretend to kick a ball” task In the initial portion of the video, the right foot performs a smooth motion resembling a ball-kicking action. In the latter portion, the left foot attempts the same task but fails to perform a kicking motion. Instead, inappropriate knee flexion and extension movements are observed.

During hospitalization, physical therapy focused on improving mobility skills with the goal of enabling discharge to the patient’s home. Interventions included gait training, stair climbing exercises, and outdoor walking practice. These rehabilitation interventions were specifically tailored to the patient’s impairments. For example, ergometer training was used to address bilateral coordination deficits, while ball-kicking and imitation tasks were selected to target lower-limb apraxia. Repetitive task-specific training and feedback-based adjustments were implemented to promote motor learning and improve functional performance.

While the uncoordinated movements observed during the foot pat task showed little improvement and persisted at discharge, the phenomenon of placing both feet on a single step during stair descent, which was frequently observed during hospitalization, was resolved by the time of discharge. Similarly, initial difficulties in achieving smooth pedaling during ergometer tasks were noted; however, by the time of discharge, the patient was able to pedal fluidly. In the task involving kicking a hemispherical ball, erroneous responses disappeared by discharge, but the clumsiness in performing the kicking motion persisted. Furthermore, in the task requiring the patient to mimic a ball-kicking motion, improvement was observed by the time of discharge. However, compared to the right lower limb, the left lower limb continued to exhibit clumsiness in movement execution. By the time of the final assessment, the patient's performance on the SPTA showed overall improvement in both upper and lower limbs, though some deficits persisted, mainly in the left upper limb (Table 1). Severe errors were limited to meaningless gesture tasks involving intermanual transfer and bimanual imitation. Mild errors remained in left-hand habitual gestures, tool-use gestures, and actual tool use, as well as in figure drawing tasks. However, compared to the initial assessment, the patient demonstrated greater accuracy and fewer severe errors, indicating a partial recovery in learned motor execution and coordination across both upper and lower limbs.

Ultimately, the patient achieved a FIM score of 101, comprising 79 points for motor function and 22 points for cognitive function, enabling discharge to her home. The authors provided the patient with a verbal explanation of the purpose of this report and obtained written informed consent for publication.

## Discussion

This case highlights two key findings: (1) bilateral lower-limb coordination deficits, providing novel evidence that the corpus callosum is critical for interhemispheric motor integration beyond the upper limbs, and (2) left lower-limb apraxia resulting from left ACA infarction, suggesting that callosal dysfunction is a primary contributor rather than frontal damage alone.

Role of the corpus callosum in bilateral lower-limb coordination

In patients with corpus callosum damage, bilateral motor tasks often exhibit timing mismatches, highlighting the importance of interhemispheric communication for coordinated motor control [5]. While callosal lesions are well known to disrupt bimanual coordination, their impact on bilateral lower-limb movements remains underreported. In the present case, a timing mismatch was observed during the foot pat task, where the patient was instructed to operate both foot pats simultaneously. Similarly, disrupted stair descent rhythm and impaired reciprocal pedaling movements suggest that the corpus callosum plays a crucial role in lower-limb motor coordination, just as it does for the upper limbs.

Rhythmic lower-limb movements, such as those required for pedaling an ergometer or level-ground walking, involve multiple motor centers, including the basal ganglia, cerebellum, brainstem, spinal cord, and cerebral cortex, which collectively send and coordinate motor commands [11]. Movement initiation in one limb is typically accompanied by the inhibition of the contralateral limb via interhemispheric communication through the corpus callosum [12]. This mechanism prevents simultaneous movement of both limbs during unilateral tasks [13]. The initiation of ergometer pedaling requires the suppression of contralateral limb movement when one limb begins pedaling. In this case, the corpus callosum lesion likely impaired this coordination, causing simultaneous movement of both limbs at the initiation of pedaling.

Similarly, the patient exhibited disrupted rhythmicity during stair descent, a pattern absent during level-ground walking. Coordination accuracy decreases as motor tasks become more complex [5], which likely explains the rhythmic disruption observed during stair descent. Recent studies using MR tractography have demonstrated dense fiber connections between the lower-limb motor areas of both hemispheres, which converge in the body of the corpus callosum [7]. In this case, the lesion involved the body of the corpus callosum, which likely affected the callosal fibers connecting motor areas of the lower limbs (Figure 1). The body contains motor fibers connecting homologous lower-limb motor cortices, and its damage likely contributed to the observed bilateral lower-limb incoordination by disrupting interhemispheric motor integration [6]. These findings strongly associate bilateral lower-limb coordination impairments with specific lesion sites in the corpus callosum.

Callosal mechanisms underlying left lower-limb apraxia

Previous studies have reported left lower-limb apraxia following right ACA infarction, suggesting that callosal dysfunction disrupts interhemispheric motor control. Ito et al. reported that lesions involving the right medial frontal lobe and the genu and body of the corpus callosum can result in impaired initiation of left lower-limb movements and produce inappropriate motor responses to verbal instructions [4]. In this case, although similar lesions were identified in the medial aspect of the left frontal lobe and the entire corpus callosum (splenium, body, and genu), the patient did not exhibit difficulty in initiating movement in the left lower limb. This contrasts with previous reports suggesting that the corpus callosum may play a more crucial role in the development of left-leg apraxia than medial frontal lesions alone.

Several hypotheses explain callosal damage-induced transmission impairment. Geschwind suggested that linguistic commands processed in the left hemisphere are not transmitted to the premotor cortex of the right hemisphere via the corpus callosum, resulting in impaired motor execution [14]. Liepmann and Maas proposed that motor engrams for skilled movements are predominantly stored in the left hemisphere of right-handed individuals, and damage to the corpus callosum prevents their transfer to the right hemisphere, causing left-limb apraxia [9]. Yamadori et al. hypothesized that intentional motor control information is not transmitted to the right hemisphere, inducing apraxia during testing [15]. Collectively, these hypotheses suggest that corpus callosum damage disrupts interhemispheric communication, impeding imitation and motor actions based on verbal instructions. Specifically, the body of the corpus callosum contains motor callosal fibers connecting homologous premotor and primary motor cortices, and damage to this region may disrupt the transmission of motor engrams from the dominant hemisphere. In this case, the patient’s left lower limb exhibited incorrect responses to both imitation and verbal instructions, suggesting that disconnection of these transcallosal pathways, particularly within the body, may have contributed to the observed ideomotor apraxia in the left upper and lower limbs.

Apraxia was observed in both the left upper and lower limbs. Ambrosoni et al. reported that ideomotor apraxia of the lower limbs is closely associated with apraxia of the upper limbs, particularly in cases of severe upper-limb apraxia [10]. In their study, 17% of patients with left hemispheric lesions exhibited lower-limb apraxia, often accompanied by extensive brain damage and severe upper-limb apraxia [10]. Therefore, the left-leg apraxia observed in this patient may reflect a broader manifestation of apraxia severity, including the left upper limb.

Recovery of left lower-limb apraxia and bilateral coordination deficits following corpus callosum damage

Previous studies have suggested that the recovery of ideomotor apraxia, particularly in the left hand, is associated with the reconstruction of callosal fibers in the posterior midbody following focal callosal damage [16]. The observed improvement in left-leg apraxia in this case may similarly be attributed to the functional reorganization of transcallosal pathways. Supporting this notion, previous work has demonstrated that comprehensive rehabilitation can induce changes in functional connectivity and promote neuroplasticity in stroke patients [17]. In our case, intensive multidisciplinary rehabilitation-including physical, occupational, and speech therapy-may have facilitated plasticity of the residual callosal fibers, contributing to motor recovery of the left lower limb. Regarding the improvement in bilateral lower-limb incoordination, it has been reported that patients with preserved callosal white matter exhibit greater responsiveness to rehabilitation programs focused on balance and coordination [7]. Furthermore, the efficacy of lower limb and trunk balance training in individuals with callosal infarction has been demonstrated [18]. Although neuroimaging data such as diffusion tensor imaging (DTI) or tractography were not available in our case, the structured gait and stair training administered during hospitalization might have played a significant role in facilitating the observed functional improvements in lower-limb coordination.

Limitations of the current case and future directions

This patient demonstrated bilateral lower-limb coordination impairments and apraxia affecting both the left upper and lower limbs, suggesting that extensive corpus callosum damage plays a critical role in these motor dysfunctions. However, this report has limitations. First, the involvement of the corpus callosum in bilateral limb coordination is not definitive, as control of these movements may also involve subcortical circuits and the supplementary motor area [19]. Further studies incorporating quantitative motor assessments and detailed investigations of lesion location and motor impairments are required [20]. A deeper understanding of these mechanisms could also provide insights into interhemispheric motor coordination deficits observed in other neurological conditions, such as multiple sclerosis and traumatic brain injury.

Second, this report is based on a single case, which is insufficient to establish a definitive relationship between the lesion site and lower-limb apraxia. Lower-limb movements generally have fewer degrees of freedom compared to upper-limb movements and are less likely to result in apraxia unless the lesion is severe [10]. Additional case reports and comparative studies are essential to clarify the impact of corpus callosum damage on lower-limb motor control. Such investigations may also help refine clinical assessments of motor dysfunction associated with callosal lesions, contributing to more precise diagnostic and therapeutic approaches. Third, this case highlights corpus callosum dysfunction in lower-limb praxis, but potential confounding factors such as cognitive function, prior motor experience, and compensatory mechanisms should be considered. Finally, while ideomotor apraxia and bilateral coordination impairments were observed, some differences in lower-limb function may reflect the inherent variability of a single case rather than true statistical significance. These findings require further validation in larger studies.

## Conclusions

In conclusion, we describe a case with unique manifestations, including left-leg apraxia and bilateral lower-limb incoordination, resulting from extensive corpus callosum damage. These findings highlight the critical role of the corpus callosum in interhemispheric communication and bilateral lower-limb motor control. Additionally, the findings indicate that the left hemisphere, as the dominant hemisphere for motor planning, may also contribute to lower-limb apraxia. However, as this is a single-case report, the findings have limited generalizability. Further research is needed to develop standardized assessments for lower-limb apraxia and bilateral coordination deficits, which remain poorly defined. Integrating neurophysiological assessments and advanced imaging techniques may help clarify the contribution of interhemispheric connections to motor coordination.

## Appendices

**Table 2:** Clinical timeline from acute to rehabilitation phase MRI: magnetic resonance imaging; FIM: functional independence measure; FAB: Frontal Assessment Battery

Days from onset	Key events	Neurological findings	Treatment and rehabilitation progress
Day 0 (onset)	Difficulty walking, slurred speech	MRI: infarctions in the corpus callosum and left frontal lobe	Antiplatelet therapy (aspirin 100 mg/day)
Day 12 (transfer to rehabilitation hospital)	Transferred to rehabilitation hospital	Mild sensory impairment, word-finding difficulty, no motor paralysis	Intensive rehabilitation started; FIM: 44
Days 20–40	Left leg apraxia and bilateral lower-limb coordination deficits persist	Frontal dysfunction (FAB: 7/18), impaired interhemispheric motor transfer	Continued rehabilitation with gait training, stair exercises, and ergometer training
Day 124 (discharge to home)	Discharged home after rehabilitation	Improved motor coordination, residual clumsiness in left lower limb	FIM: 101; able to walk independently
